# Theory of Mind and Social Informant Discrepancy in Autism

**DOI:** 10.1007/s10578-024-01676-4

**Published:** 2024-03-19

**Authors:** Alister S. Collins, Kevin J. Carroll, Alan H. Gerber, Elliot Gavin Keenan, Matthew D. Lerner

**Affiliations:** 1 Renaissance School of Medicine, Stony Brook University, Stony Brook, USA; 2 Department of Psychology, Stony Brook University, Stony Brook, USA; 3 Yale Child Study Center, Yale University School of Medicine, New Haven, USA; 4 Social Connection and Treatment Lab, Life Course Outcomes Research Program, AJ Drexel Autism Institute, Drexel University, 3020 Market Street, #560, Philadelphia, PA 19104 USA

**Keywords:** Autism, Theory of mind, Social skills, Informant discrepancy, Polynomial regression

## Abstract

When autistic youth are asked to assess their own social skills, they frequently rate themselves more favorably than their parents rate them. The magnitude of this informant discrepancy has been shown to relate to key clinical outcomes such as treatment response. It has been proposed that this discrepancy arises from difficulties with Theory of Mind. Participants were 167 youth 11 to 17 years old; 72% male, and their parents. Youth completed self-report measures of social skills and social cognitive tasks, while their parents completed questionnaires regarding social skills. A repeated-measures ANOVA indicated both non-autistic and autistic youth rated themselves more favorably than their parents rated them across all measures. Zero-order correlations revealed that raw differences between parent- and participant-report were negatively correlated with scores on parent-reported Theory of Mind measures. However, polynomial analysis did not indicate interaction effects between parent- and participant-report on any of the measures used. Polynomial regression revealed that increases in parent-reported social skill predicted larger increases in parent-report Theory of Mind at low levels of parent-reported social skill compared to high levels of parent-reported social skill. Participant-report social skills predicted performance on a behavioral Theory of Mind test in a curvilinear fashion, such that the relationship was positive at low levels of participant-reported social skills, but *negative* at high levels. This study replicates the finding that raw difference score analyses may result in illusory effects that are not supported when using more contemporary analysis methods, and that more complex and subtle relationships between social insight and perspective-taking exist within autistic youth.

Autism is a neurodevelopmental condition characterized by differences in social and emotional reciprocity, nonverbal communication, and relationship formation [1]. As such, autistic individuals may view their own social abilities differently than others view them [2]. Social informant discrepancy is defined as the difference between self-report and parent (or other reporter) evaluations of a child’s social skills. When autistic adolescents are asked to assess their own social skills, their self-assessed ratings are often higher than their parents’ assessments of their social skills—that is, they exhibit a pronounced social informant discrepancy. Little is known, however, about what social cognitive processes may underlie such discrepancies in this population. One candidate is Theory of Mind—or the ability to understand others’ perspectives. While recent work shows conclusively that autistic individuals do *not* show a characteristic Theory of Mind deficit [3], it is nonetheless the case that there are individual differences in Theory of Mind among autistic people, and such differences may relate meaningfully to other social outcomes [4, 5]. While social informant discrepancies may represent such an outcome, much work in this field has been hindered by use of imprecise—or even inaccurate—analytic methods for quantifying them [6]. Utilizing more quantitatively precise methods for measuring informant discrepancy in relation to Theory of Mind would allow for better specification of the relationship between these two key social constructs among autistic people, and would allow practitioners to make more informed treatment decisions based on the information they received; it would also allow researchers to have clearer hypotheses and methods for advancing this crucial area of study for this population.

## Parent–Child Social Informant Discrepancy

In practice, clinicians often receive reports from multiple informants. For example, an assessment might include reports from a child’s parents, their teacher, and the child themselves. No single measure can completely capture an individual’s mental health [7], indicating that single-informant reports are limited and do not capture information, such as clinical outcomes, that can be captured by examining the reports of multiple informants. Measuring *informant discrepancy*, or the difference between one informant’s report of a given quality (such as social skill) and another informant’s report of that same quality is an important method for interpreting multiple informants’ reports. This parent–child informant discrepancy is one of the most frequently studied types of informant discrepancy and has been found to predict clinical outcomes for socially impaired populations [8]. This discrepancy is particularly pronounced in some clinical populations. For example, youth with ADHD frequently report less social impairment compared to parents’ assessment of their social skills [9]. In general-population samples of youth, youth often report greater-than-average social skills, although the size of this effect is mixed and often small [10, 11].

## Informant Discrepancy in Autistic Individuals

There is a small but growing body of research on social informant discrepancy in autistic individuals. Lerner et al. [12] investigated discrepancies between parent-reported and child-reported social functioning in autistic adolescents with low or moderate support needs. Their findings indicated that greater discrepancy predicted lower hostile attribution to peers in ambiguous social scenarios, less depression, as well as significant decreases in self-reported social anxiety during a summer social skills intervention. Results also indicated that social informant discrepancy (using raw difference scores) predicted clinical outcomes such as parental self-efficacy, depression, social anxiety, and youth-reported hostile attributions to peers. Social informant discrepancy observed in this study was approximately one standard deviation [12]. Another study examined social informant discrepancy in autistic youth and found a discrepancy of slightly more than one standard deviation [13]. In both studies, this effect appears to be driven by parents rating the social competence of their children much lower than the children rated themselves. When autistic adolescents are asked to assess their own social skills, their self-assessed ratings are often higher than their parents’ assessments of their social skills—that is, they exhibit a pronounced social informant discrepancy [12, 14].

## Theory of Mind & Social Informant Discrepancy

Theory of Mind is defined as the ability to model the mental states of others. While Theory of Mind challenges are not a core characteristic of autism [3], differences in Theory of Mind have frequently been observed between autistic and non-autistic people and these differences have been widely studied for the last forty years. Further, Theory of Mind abilities have been shown to predict outcomes such as social skill, support needs, and restrictive and repetitive behaviors. [4]. Part of this is likely due to the double-empathy problem, or the concept that autistic individuals can accurately read the mental states of other autistic individuals, but find it challenging to read the mental states and infer the motivations of non-autistic people [15]. Importantly, the double-empathy problem also extends to non-autistic people reading the mental states of autistic individuals. This makes Theory of Mind a potentially valuable predictor of clinical outcomes for autistic individuals. Specifically, an individual with a poor ability to model the mental state of others may also be poor at understanding how others perceive them in social situations. This may lead to an inflated or inaccurate estimate of social performance.

Research examining Theory of Mind and informant discrepancies in autistic populations is limited. No work has specifically examined if, and for what social skills domains, informant discrepancies relate to Theory of Mind. Only one study has examined Theory of Mind and social informant discrepancies in autism, finding that for both mentalistic and non-mentalistic aspects of social behavior, parents gave more positive reports than professionals [16]. Crucially, much of this work has not leveraged recent advances in multi-informant assessment of Theory of Mind, such as task-based versus informant-based assessments [17]. Thus, there is a need to further examine the relationship between social informant discrepancies and Theory of Mind in autistic youth.

## Methodological Issues with Informant Discrepancy Measurement

The use of raw differences scores to understand informant discrepancy, while useful in some limited ways, have important shortcomings which are important for researchers and clinicians to understand. Comparison of widely used shorthand methods such as comparison of raw difference scores to more rigorous, accurate approaches such as polynomial regression allows a better understanding of how traditional methods are misleading, as well as a deeper understanding of the dynamics of extant relations between such variables. Past research has approached informant discrepancies using a raw difference score method, taking the raw difference between child-reported scores on a measure and the parent-reported scores. Difference scores have methodological issues associated with them, such as failing to capture higher-order effects on variables as well as being mathematically constrained. If difference scores are being computed for a third variable (such as Theory of Mind) from two other variables (such as parent reported social skill and child reported social skill) the correlation between that difference score is entirely explained by the variance and correlation of the individual measures [18]. In addition, measuring raw informant difference scores constrains variables: a model that measures the effect on a dependent variable using the raw difference between two independent variables constrains the size of effects on these independent variables to be equal in magnitude and opposite in sign. This limitation means that this type of model fails to capture differences in the size of contributions to a dependent variable; it also does not capture moderation effects or quadratic effects.

Polynomial analysis is capable of capturing these differences: a model that tests parent (P) and participant (A) informant discrepancy hypotheses using the equation *Y* = *b*_*0*_ + *b*_*1*_*P* + *b*_*2*_*A* + *b*_*3*_P*A + *b*_*4*_*P*^*2*^ + *b*_*5*_*A*^*2*^ is able to capture both linear and quadratic effects of parent and child-reported variables. The P*A interaction term tests whether any association between participant reports and outcome is moderated by the parent report; it can also be interpreted in the opposite direction to test whether an association between parent reports and outcome is moderated by participant report. In addition, polynomial analysis is not subject to the same mathematical constraints as raw difference scores.

Crucially, to date, nearly all studies relating social informant discrepancy to any social cognitive outcome (e.g., Theory of Mind), whether in autistic or non-autistic youth, have utilized raw, standardized, or residualized difference scores—in other words, they did not use the more precise polynomial regression approach. In one of the few exceptions [18], in a sample of non-autistic adolescents, poorer task-based emotion recognition abilities (among children and parents) related to greater perceived discrepancies regarding beliefs about daily life tasks, but the nature of the relationship was subtler than previous research suggested, indicating that difference score-based approaches may artificially inflate such relationships. However, this study did not use polynomial regression analysis, nor did it use children’s self-reports. Thus, it may be that more direct assessment of social informant discrepancies in relation to multi-method assessment of Theory of Mind, utilizing contemporary polynomial regression-based analyses, may reveal that these relations may be attenuated, or more complex (e.g., accounted for by higher-order polynomial relationships) than previously suggested in the literature. Such a result would be important for advancing understanding of theory of mind and informant discrepancy in autism.

## Present Study

The current study seeks to address the gaps in this research by investigating informant discrepancies utilizing a more rigorous statistical approach (i.e., polynomial regression). Specifically, we investigated the relationship between Theory of Mind and discrepancies in participant and parent report on two measures of social functioning: a parent-reported Theory of Mind questionnaire and a task-based Theory of Mind activity. We hypothesized that (1a) participants would rate their social functioning significantly higher than their parents rate their social functioning. We then hypothesized that (1b) the raw difference between participant-rated social functioning and parent-rated social functioning would be larger for the autistic group. We hypothesized that (2) parent-participant social informant discrepancy would relate to Theory of Mind (parent-rated and task-based), after controlling for autism severity, using traditional raw discrepancy analyses. Finally, we hypothesized that (3) more rigorous, contemporary analytic approaches would reveal differences in the relationship between social informant discrepancy and Theory of Mind compared to those seen using more contemporary analytic approaches.

## Methods

### Participants

The sample consisted of 106 autistic and 61 non-autistic adolescents (age range = 11–17; see Table 1) who were participating in a larger study of social behavior. Inclusion criteria for the larger study included IQ > 70 on the Kaufman Brief Intelligence Test (KBIT-2; Kaufman, 2004). Participants were also required to have no significant cognitive deficits including severe medical or psychiatric impairments or comorbidities that would impede completion of study activities. All autism diagnoses were confirmed with an ADOS-2 assessment conducted by research-reliable examiners.

**Table 1:** Demographic and descriptive characteristics of the sample

Variable	*N* (%)	Autistic (*n* = 106)	Non-autistic (*n* = 61)	*p-*value^a^
Male (%)	119 (71.7%)	84 (79.2%)	36 (59.0%)	0.005
Race/ethnicity (%)				
White/Caucasian	144 (86.2%)	53 (86.9%)	91 (85.8%)	0.852
African American/Black	6 (3.6%)	2 (3.3%)	4 (3.8%)	0.869
Asian/Asian American	7 (4.2%)	2 (3.3%)	5 (4.7%)	0.655
Hispanic/Latino	16 (9.6%)	9 (14.8%)	7 (6.6%)	0.085
Native American/American Indian or Alaskan native	3 (1.8%)	0 (0.0%)	3 (2.8%)	0.185
Native Hawaiian or Other Pacific Islander	1 (0.6%)	1 (1.6%)	0 (0.0%)	0.186
Other	3 (1.8%)	0 (0.0%)	3 (2.8%)	0.185
Declined to answer	3 (1.8%)	1 (1.6%)	2 (1.9%)	0.908
Family income (%)				0.508
< $15,000	4 (2.4%)	1 (1.6%)	3 (1.8%)	
$15,000–$30,000	6 (3.6%)	1 (1.6%)	5 (3.0%)	
$30,000–$45,000	6 (3.6%)	3 (4.9%)	3 (1.8%)	
$45,000–$60,000	7 (4.2%)	4 (6.6%)	3 (1.8%)	
$60,000–$75,000	4 (2.4%)	2 (3.3%)	2 (1.2%)	
$75,000–$90,000	19 (11.4%)	6 (9.8%)	13 (7.8%)	
$90,000–$105,000	14 (8.4%)	7 (11.5%)	7 (4.2%)	
$105,000–$120,000	18 (10.8%)	7 (11.5%)	11 (6.6%)	
$120,000–$135,000	12 (7.2%)	4 (6.6%)	8 (4.8%)	
$135,000–$150,000	7 (4.2%)	5 (8.2%)	2 (1.2%)	
$150,000–$165,000	8 (4.8%)	3 (4.9%)	5 (3.0%)	
$165,000–$180,000	10 (6.0%)	5 (8.2%)	5 (3.0%)	
> $180,000	34 (20.4%)	9 (14.8%)	25 (15.0%)	
Declined to answer	18 (10.8%)	4 (6.6%)	14 (8.4%)	

Variable	*Mean* (*SD*)	Autistic (*n* = 106)	Non-autistic (*n* = 61)	*p-*value^b^
Age (in years)	13.83 (1.98)	13.55 (1.87)	13.99 (2.03)	0.164
IQ (KBIT-2)	102.99 (15.87)	100.77 (16.28)	106.85 (14.39)	0.017
ADOS-2 Comparison Score	5.61 (3.24)	7.74 (1.85)	1.92 (0.86)	< .001
ToMI-2	15.78 (3.45)	15.22 (3.20)	16.54 (4.08)	0.005
SELWeb Theory of Mind^c^	88.22 (14.76)	85.91 (17.13)	92.22 (8.02)	0.009
SSiS-RS—Participant^d^	95.23 (16.17)	93.63 (16.21)	98.07 (15.84)	0.09
SSiS-RS—Parent	85.43 (18.43)	81.90 (16.69)	91.57 (19.78)	0.001
SPP-A—Close Friendship—Participant^e^	2.62 (0.72)	2.63 (0.69)	2.62 (0.82)	0.95
SPP-A—Close Friendship—Parent^f^	1.90 (1.05)	1.69 (0.94)	2.34 (1.15)	0.003
SPP-A—Social Competence—Participant	2.56 (0.76)	2.51 (0.73)	2.48 (0.79)	0.191
SPP-A—Social Competence—Parent^g^	1.98 (0.92)	1.72 (0.73)	2.66 (0.82)	< .001

*KBIT-2* Kaufman brief intelligence test, 2nd Edition; *ADOS-2* autism diagnostic observation schedule, Second Edition; *ToMI-2* theory of mind inventory-2; *SELweb Theory of Mind* social reasoning subtest from SELweb (Social-Emotional Learning) web activity; *SSiS-RS* social skills improvement system—rating scales; *SPP-A* self-perception profile for adolescents

^a^*p*-values for discrete variables calculated using *χ*^*2*^ tests

^b^*p*-values for continuous variables calculated using two-sample *t* tests

^c^*N* = 161

^d^*N* = 166

^e^*N* = 98

^f^*N* = 102

^g^N = 163

### Measures

*The Autism Diagnostic Observation Schedule, Second Edition* (ADOS-2) [19]. The ADOS-2 is a semi-structured, standardized observational instrument that assesses social and communicative abilities of individuals through a series of prompts. The ADOS-2 is considered a gold-standard assessment for diagnosing autism. The ADOS-2 produces a diagnostic classification of autism, autism spectrum, or non-spectrum. Participants were considered autistic for the purposes of our analyses if they scored in the autism or autism spectrum range. All study participants received an ADOS-2 assessment. All ADOS-2 assessments were completed by research-reliable assessors.

*Kaufman Brief Intelligence Test 2nd Edition* (KBIT-2) [20].The KBIT-2 is a brief measure of intelligence for use in children and adults over the age of 4 years. It provides a verbal IQ score, a nonverbal IQ score, and an overall composite score.

*Social Skills Improvement System—Rating Scales* (SSiS-RS) [21]. The SSiS-RS is a measure designed to evaluate social skills across typical and clinical populations. It is designed to be administered in parallel, giving normed evaluations of parent-reported, teacher-reported, and self-reported social skills as defined by the same constructs. The SSiS-RS assesses three domains: social skills, problem behaviors, and academic competence. It consists of 79 items in the parent form and 75 items in the child form, with each item (e.g., “I say please when I ask for things”) rated on a 4-point Likert scale (from “0—not true” to “3—very true”). The SSiS-RS produces normed standard scores with a mean of 100 and a standard deviation of 15. Higher scores reflect greater social skills. For this study, only the social skills subscale was used. The SSiS-RS has been widely used and evinced adequate psychometric properties in autistic children and adolescents [22]. Internal consistency (Cronbach’s α) for the parent- (0.96) and child self-report (0.95) SSiS-RS for this sample was high.

*Self-Perception Profile for Adolescents, Parent Edition & Child Edition* (SPP-A) [23]The SPP-A is a parent- and child-reported measure of self-worth. The two domains of interest used in this study are parent report of Social Competence and Close Friendship, and the equivalent child subscales (Self-Perception of Social Competence and Self-Perception of Close Friendship). The SPP-A poses questions in a *structured alternative format* [24] in which the survey taker is asked to first decide which option best describes them or their child: the one on the left or the one on the right. After this decision is made, the individual then decides whether the description is “Really True” or “Sort of True”. For example, a question about friendship would consist of a prompt “This individual is able to make close friends OR this individual finds it hard to make really close friends.” For the “This individual is able to make close friends” prompt (on the left side), there is a “really true” and a “sort of true” option. The same “really true” and “sort of true” options are available for the “This individual finds it hard to make really close friends” prompt on the right side; both opposing prompts are part of the same question. Thus, there are four possible answers for each question, and the scale is a four-point scale. Total subscale scores are calculated by averaging the five items in each subscale, with a total score ranging from 1 to 4. The SPP-A has been utilized with autistic youth in prior research [25].

*Theory of Mind Inventory-2* (ToMI-2) [17]. The ToMI-2 is a 60-item parent-report questionnaire that measures Theory of Mind functioning as expressed in real-world samples of behavior. Each item takes the form of a statement (e.g., “My child understands whether someone hurts another on purpose or by accident”) and a 20-point sliding continuum anchored by ‘definitely not’, ‘probably not’, ‘undecided’, ‘probably’, and ‘definitely.’ Internal consistency for the ToMI-2 for this sample was high, Cronbach’s α = 0.97. The ToMI-2 has been widely used and evinced adequate psychometric properties in both nonautistic and autistic participants [17, 26].

*SELweb Theory of Mind* [27]. We used the social reasoning subtest (Theory of Mind) from the SELweb assessment. SELweb is a computer-based assessment that assesses various dimensions of social-emotional comprehension. The social reasoning (Theory of Mind) subtest presents illustrated and narrated vignettes of social situations for which children are asked to answer questions in which they must infer the characters’ mental or emotional states. There are 36 total questions in this subtest, which has been used with autistic children, and shown evidence of reliability and validity in these samples [28, 29].

### Procedure

The dataset for the current study was extracted from a larger study focused on social interactions of autistic and non-autistic adolescents. Informed consent and assent were obtained from parents and participants prior to all data collection. All participants were administered an ADOS-2 and a KBIT-2 to verify their eligibility for the study activities. ADOS-2 assessments were completed by research-reliable administrators. Participants completed the child/adolescent versions of the SSiS-RS, SPP-A, and SELweb Theory of Mind during one of the lab visits associated with the larger study. Parents of participants completed the parent versions of the SSiS-RS, SPP-A, and ToMI-2 measures during the same visit.

### Data Analytic Plan

All statistical analyses were conducted on SPSS Statistics Version 27.0. First, we obtained descriptive statistics on the sample. Next, we ran correlations to assess the relationships among relevant clinical variables.

To test Hypothesis 1, that participants would rate their social functioning significantly higher than their parents rate their social functioning and that there would be a larger difference between participant-reported and parent-reported social functioning for autistic participants, we compared parent-reported scores on the SSiS-RS and two SPP-A subscales with participant-reported scores on the same measures. We ran a repeated-measures Analysis-of-Variance test (ANOVA) on parent reported and participant reported SSiS-RS, SPP-A Close Friendship, and SPP-A Social Competence, with informant (parent vs. participant) as the within-subjects factor, and diagnostic status (autistic vs. non-autistic) as the between-subjects factor.

To investigate Hypothesis 2, that parent-participant social informant discrepancy would predict Theory of Mind (ToMI-2 and SELweb Theory of Mind) scores controlling for autism severity, we first calculated raw difference scores by subtracting participant ratings from parent ratings. Thus, the raw difference score reflects how much greater participants rated their social functioning than their parents rated them. We ran zero-order correlations between raw difference scores on the SSiS-RS, SPP-A Social Competence, and SPP-A Close Friendship and Theory of Mind (ToMI-2 and SELweb ToM) scores.

To examine Hypothesis 3, that polynomial analysis would predict the effect of parent-reported and participant-reported measures of social functioning on Theory of Mind Inventory scores and SELweb Theory of Mind scores in ways that linear analysis would not, we conducted polynomial regression analysis using either parent-report Theory of Mind Inventory-2 (ToMI-2) or task-based (SELweb Theory of Mind) as dependent variables. The polynomial regression model with moderation for testing participant (A) and parent (P) informant discrepancy hypotheses (as a predictor of Y), while controlling for a covariate, in this case ADOS-2 Comparison Score (W) is shown as follows: $Y={b}_{0}+{b}_{1}A+{b}_{2}P+{b}_{3}{A}^{2}+{b}_{4}AP+{b}_{5}{P}^{2}+{b}_{6}W$. SSiS-RS, SPP-A Close Friendship, or SPP-A Social Competence were the independent variables. For the polynomial regression model, we ran two sets of three analyses. The first set of analyses were regression analyses with parent ToMI-2 scores as the dependent variable, the parent measure (SSiS-RS, SPP-A Social Competence, and SPP-A Close Friendship) as the independent variable, and the participant measure (SSiS-RS, SPP-A Social Competence, and SPP-A Close Friendship) as moderators; squared parent and participant measures were also included to account for potential higher order effects. ADOS-2 comparison score was the covariate. The dependent variable in the second set of analyses was SELweb Theory of Mind. Polynomial regression models were calculated in accordance with guidelines recommended by Laird & De Los Reyes [18].

## Results

The autistic and non-autistic groups differed in terms of IQ (lower IQ in the autistic group), sex (more males in the autistic group), and ADOS-2 scores (higher in the autistic group), as well as parent-reported ToMI-2, SELWeb Theory of Mind, SSiS-RS and SPP-A scores (lower in autistic group). They did not differ in terms of self-reported SSiS-RS and SPP-A scores.

### Hypothesis 1

Autism in relation to difference between participant- and parent-report.

For the SSiS-RS, there was a significant main effect of informant on SSiS-RS score, such that participants rated themselves higher than their parents rated them. There was also a significant main effect of autism diagnostic status on SSiS-RS score, such that autistic participants received lower scores than non-autistic participants. There was no significant interaction between informant status and autism (Table 2).

**Table 2:** Repeated-measures ANOVA depicting main effect of informant and participant, and informant × participant interaction

Measure	Effect	*df*	*F*	*p*	*B (SE)*	Partial *η2*	Independent variables	*Mean (95% CI)*
SSiS-RS	Autism status	164	11.33	0.001	6.95 (2.07)	0.07	Autistic	87.76 (85.31–90.22)
							Non-autistic	94.72 (91.45–97.98)
	Informant		25.82	< 0.001	9.22 (1.81)	0.14	Parent	86.63 (83.78–89.49)
							Participant	95.85 (93.29–98.41)
	Autism status × informant		1.93	0.17	–	0.01	Autistic × parent	81.90 (78.46–85.33)
							Autistic × participant	93.63 (90.55–96.72)
							Non-autistic × parent	91.37 (86.80–95.93)
							Non-autistic × participant	98.07 (93.97–102.16)
SPP-A SC	Autism status	161	15.89	< 0.001	0.41 (0.10)	0.09	Autistic	2.12 (2.00–2.24)
							Non-autistic	2.53 (2.37–2.70)
	Informant		36.96	< 0.001	0.50 (0.08)	0.19	Parent	2.08 (1.94–2.22)
							Participant	2.57 (2.45–2.69)
	Autism status × informant		13.91	< 0.001		0.08	Autistic × parent	1.72 (1.55–1.89)
							Autistic × participant	2.52 (2.38–2.66)
							Non-autistic × parent	2.44 (2.21–2.66)
							Non-autistic × participant	2.63 (2.43–2.82)
SPP-A CF	Autism status	95	4.05	0.047	0.30 (0.15)	0.04	Autistic	2.16 (1.99–2.32)
							Non-autistic	2.45 (2.21–2.69)
	Informant		26.61	< 0.001	0.64 (0.12)	0.22	Parent	1.98 (1.77–2.20)
							Participant	2.62 (2.46–2.78)
	Autism status × informant		5.92	< 0.001	–	0.06	Autistic × parent	1.69 (1.44–1.93)
							Autistic × participant	2.62 (2.45–2.80)
							Non-autistic × parent	2.28 (1.92–2.64)
							Non-autistic × participant	2.62 (2.35–2.89)

Autism status (i.e., met inclusion criteria to be in the autism group or not)

*SSiS-RS* social skills improvement system—rating scales, *SPP-A SC* self-perception profile social competence, *SPP-A CF* self-perception profile close friendship

For SPP-A Social Competence, there was a significant main effect of informant on SPP-A Social Competence score, such that participants rated themselves higher than their parents rated them. There was also a significant main effect of autism diagnostic status on SPP-A Social Competence score, such that autistic participants received lower scores than non-autistic participants. There was a significant interaction between informant status and autism. This effect was driven by parent-reported SPP-A Social Competence in the non-autistic group being higher than in the autistic group.

For SPP-A Close Friendship, there was a significant main effect of informant on SPP-A Close Friendship score, such that participants rated themselves higher than their parents rated them. There was also a significant main effect of autism diagnostic status on SPP-A Close Friendship score, such that autistic participants received lower scores than non-autistic participants. There was a significant interaction between informant status and autism. This effect was driven by parent-reported SPP-A Close Friendship in the non-autistic group being higher than in the autistic group (Table 2).

### Hypothesis 2

Raw difference scores and theory of mind measures.

There were negative correlations between parent-report ToMI-2 and raw parent-participant differences in SSiS-RS, SPP-A Close Friendship, and SPP-A Social Competence (Table 3). There were no relationships between Theory of Mind as measured by the SELweb Theory of Mind task and discrepancies in SSiS-RS, SPP-A Close Friendship, or SPP-A Social Competence.

**Table 3:** Correlations between parent-report Theory of Mind and raw differences between parent-reported and participant-reported SSiS-RS, SPP-A Social Competence, and SPP-A Close Friendship

Variable	1	2	3	4	5	6
1. ToMI-2	–					
2. SELweb theory of mind	0.23**	–				
3. ADOS-2 comparison score	− 0.26**	− 0.28**	–			
4. SSiS-RS standard score raw difference	− 0.47**	− 0.07	0.12	–		
5. SPP-A social competence raw difference	− 0.29**	0.05	− 0.27**	− 0.46**	–	
6. SPP-A close friendship raw difference	− 0.37**	0.16	− 0.26**	− 0.43**	0.62**	–

Raw difference = Difference between parent and participant report on the given measure (participant minus parent)

*ADOS-2* autism diagnostic observation schedule, Second Edition; *ToMI-2* parent-report theory of mind inventory-2; *SELweb Theory of Mind* social reasoning subtest from SELweb (social-emotional learning) online activity; *SSiS-RS* social skills improvement system—rating scales; *SPP-A* self-perception profile

*p < .05, **p < .01

### Hypothesis 3

Polynomial analyses as models of informant discrepancy.

Polynomial regression analyses revealed that parent ToMI-2 score was predicted, in each respective model, by parent-reported SSiS-RS, SPP-A Social Competence, and SPP-A Close Friendship when controlling for ADOS-2 comparison score, but not by the interaction between parent- and participant-report in any of these three models (Table 4).

**Table 4:** Parent and participant reports of social functioning and social behavior as predictors of parent-report Theory of Mind Inventory score

Outcome	Parent-report theory of mind inventory score								
Measure	SSiS-RS			SPP social competence			SPP close friendship		
	*b*	*SE*	*p*	*b*	*SE*	*p*	*b*	*SE*	*p*
Intercept	16.4189	0.2905	< 0.001	15.4430	0.4340	< 0.001	15.7866	0.6076	< 0.001
Parent	0.1098	0.0119	< 0.001	1.2239	0.3409	< 0.001	1.4213	0.4480	0.0021
Participant	− 0.0035	0.0135	0.7924	− 0.2063	0.3553	0.5623	− 0.1077	0.4507	0.8116
Parent × participant	0.0014	0.0007	0.0553	0.2440	0.4026	0.5454	0.7171	0.5126	0.1652
ADOS-2 Comparison score	− 0.0938	0.0680	0.1699	− 0.1175	0.0861	0.1744	− 0.0445	0.1092	0.6848
Parent^2^	− 0.0013	0.0005	0.0068	− 0.1267	0.3653	0.7291	− 0.0097	0.3744	0.9793
Participant^2^	− 0.0011	0.0006	0.0872	0.6813	0.3880	0.0811	0.1169	0.5338	0.8272

Model	R^2^	*F* (6, 159)	*p*	R^2^	*F* (6, 156)	*p*	R^2^	*F* (6, 90)	*P*
	0.4434	21.113	< 0.001	0.4027	5.033	< 0.001	0.2265	4.392	0.001

*ADOS-2* autism diagnostic observation schedule, Second Edition; *SSiS-RS* social skills improvement system—rating scales; *SPP-A* self-perception profile; *SE* standard error

*p < .05, **p < .01, ***p < .001

Polynomial regression analyses revealed that SELweb Theory of Mind score was predicted by only participant SSiS-RS^2^ when controlling for ADOS-2 comparison score, but not by the interaction between parent- and participant- report. SELweb Theory of Mind was predicted by ADOS-2 score in all models. SELweb Theory of Mind score was also predicted by participant SSiS-RS^2^ (Table 5).

**Table 5:** Parent and participant reports of social functioning and social behavior as predictors of parent-report SELweb theory of mind score

Outcome	SELweb theory of mind score								
Measure	SSiS-RS			SPP social competence			SPP close friendship		
	*b*	*SE*	*p*	*b*	*SE*	*p*	*b*	*SE*	*p*
Intercept	88.9739	1.5850	< 0.001	86.5568	1.9842	< 0.001	87.7414	2.8425	< 0.001
Parent	0.0228	0.0664	0.7312	− 1.5015	1.5439	0.3323	1.0567	2.0801	0.6127
Participant	0.0238	0.0752	0.7518	− 0.0867	1.6457	0.9581	− 1.1698	2.0868	0.5765
Parent × participant	0.0031	0.0040	0.4388	1.0729	1.8400	0.5607	1.8216	2.3600	0.4423
ADOS-2 Comparison score	− 1.0979	0.3737	0.0038	− 1.2128	0.3935	0.0024	− 1.2868	0.5055	0.0127
Parent^2^	0.0034	0.0026	0.2050	1.6901	1.6590	0.3100	− 0.1954	1.7442	0.9111
Participant^2^	− 0.0077	0.0034	0.0256	− 0.0937	1.8068	0.9587	2.5886	2.4520	0.2940

Model R^2^	R^2^	*F* (6, 153)	*p*	R^2^	*F* (6, 150)	*p*	R^2^	*F* (6, 88)	*P*
	0.1199	3.474	0.030	0.0889	2.438	0.028	0.1347	2.2822	0.042

*SELweb Theory of Mind* social reasoning subtest from SELweb (social-emotional learning) online activity; *ADOS-2* autism diagnostic observation schedule, Second Edition *SSiS-RS* social skills improvement system—rating scales; *SPP-A* self-perception profile; *SE* standard error

*p < .05, **p < .01, ***p < .001

## Discussion

This was the among the first studies to directly examine the relationship between informant discrepancy in social skills and Theory of Mind in autistic youth, and the first to leverage contemporary analytical approaches. Results indicated that self-report of social skills was higher than parent-report, and that autistic youth received lower reports of their social skills than non-autistic youth, with this difference often being driven by their parent’s report. Further, while raw difference scores suggested the appearance of a relationship between social skills informant discrepancies and parent-reported Theory of Mind, polynomial regression models revealed this relationship to be illusory. Instead, more complex quadratic models better explained the relationship between specific informants’ reports of social skills and Theory of Mind across multiple measures. These results both stand in contrast to and build upon previous studies utilizing raw difference scores [12].

**Fig. 1:**
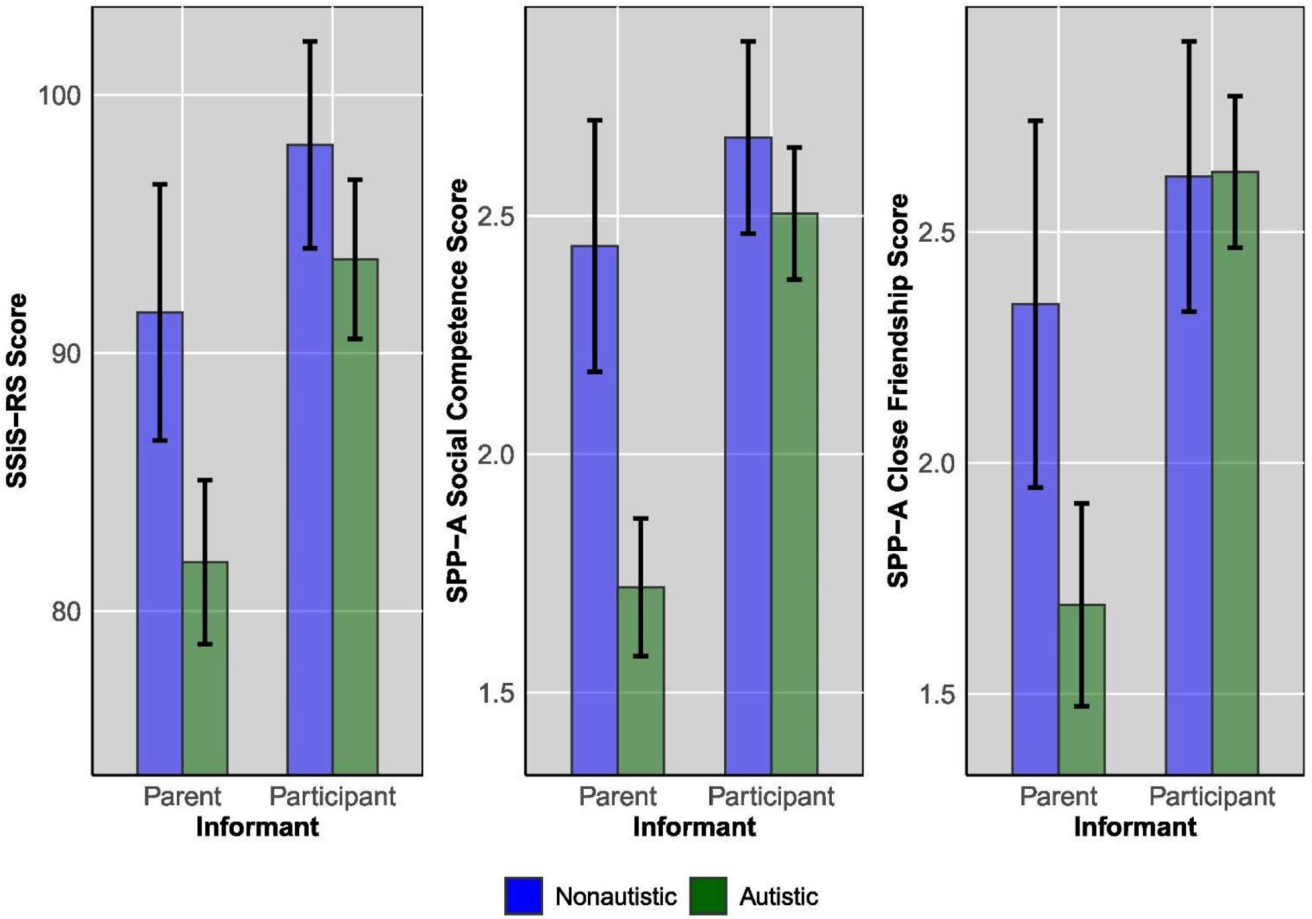
Means of parent-report and participant-report scores for measures of social behavior, by autism diagnostic status. Note: SSiS-RS: Social Skills Improvement System—Rating Scales (Standard Scores). SPP-A: Self-Perception Profile for Adolescents (Raw Scores). Error bars represent 95% confidence intervals

**Fig. 2:**
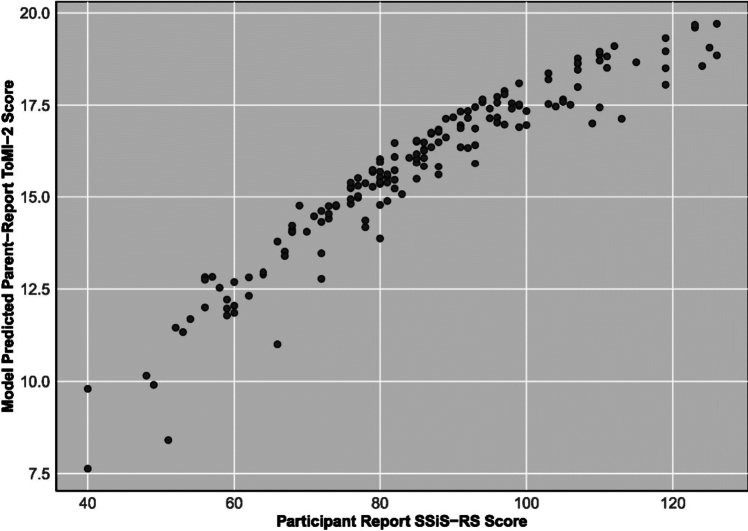
Model-predicted values of parent- report Theory of Mind Inventory-2 score by parent-report SSiS-RS score. Note. The x-axis shows model-predicted parent-reported Theory of Mind Inventory-2 score using linear and squared parent- and child- reported SSiS-RS (Social Skills Improvement System—Rating Scales) score, controlling for Autism Diagnostic Observation Schedule-2 comparison score, with linear participant-reported SSiS-RS score as moderator. Actual participant-report SSiS-RS score is on the y-axis. Parent-report not mean-centered here for visualization purposes. It is mean centered in the regression models. The corresponding statistical model can be found in Table 4. Note that the squared parent-report SSiS-RS variable is significant in this model and represented here

**Fig. 3:**
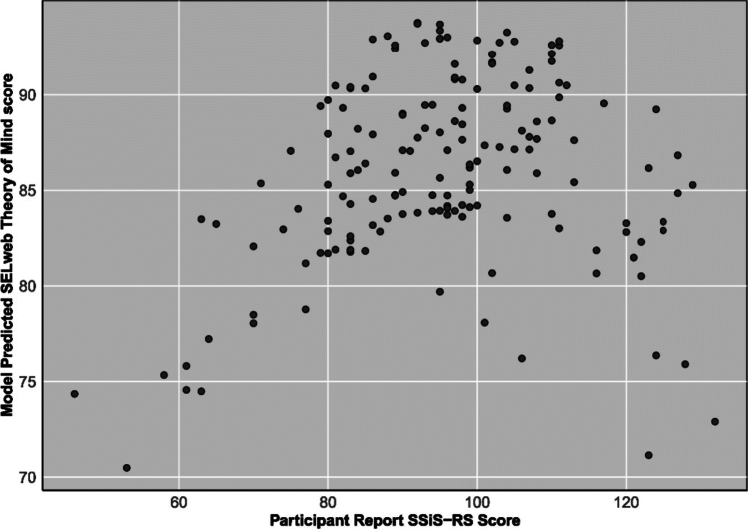
Model-predicted values of task-based SELweb Theory of Mind score by participant-report SSiS-RS score. Note. The x-axis shows model-predicted task-based SELweb Theory of Mind score using linear and squared parent- and child- reported SSiS-RS (Social Skills Improvement System—Rating Scales) score, controlling for Autism Diagnostic Observation Schedule-2 comparison score, with linear participant-reported SSiS-RS score as moderator. Actual participant-report SSiS-RS score is on the y-axis. Participant-report not mean-centered here for visualization purposes. It is mean centered in the regression models. The corresponding statistical model can be found in Table 5. Note that the squared participant-report SSIS variable is significant in this model and represented here

Consistent with our hypotheses, there was a larger raw difference between participant-rated social functioning and parent-rated social functioning as measured by SPP-A Social Competence and SPP-A Close Friendship in the autistic group. This was true even in the face of autistic youth being rated as, on average, less socially capable than their non-autistic peers across measures. This difference was due to parent-report being approximately a standard deviation lower in the autistic group, while autistic adolescents’ self-report on these measures was comparable to non-autistic adolescents’ self-report on these measures. Most straightforwardly, this may be due to parents of autistic youth noticing their children struggling socially and identifying those challenges. However, this difference could also arise due to autistic youth and their parents having fundamentally different views of which social skills are important, and what counts as successful social skill [2]. This possibility is crucial for clinicians to acknowledge and account for when assessing autistic youth and considering treatment options (Figs. 1, 2, 3).

Further, raw difference scores in social skills correlated with parent-report Theory of Mind, but not behavioral Theory of Mind, for all social competence measures. This result highlights the importance of considering informant effects in measurement. Frequently, parent reports of a measure, such as social skill, will be moderately or strongly correlated with parent reports of other closely related measures such as autism symptom severity [30, 31]. In this case it is possible that the observed relationship may simply reflect that parents who report their children to have better Theory of Mind also report them to be more socially skilled. However, the correlation between parent-reported and clinician-observed measures is often much weaker [32, 33]. This does not invalidate the clinical and research utility of parent-reported data. Instead, this highlights that parent-report is telling us something specific about the context of measurement (e.g., Theory of Mind as observed in vivo rather than captured in a lab), the informant themselves (e.g., a parent’s pattern of perception), or a pattern of perception about the child—such as seeing the child as skilled or not [34, 35].

Polynomial analysis provides an alternative and methodologically sound approach to interpreting the relationship between informant discrepancy and other measures compared to using raw scores. This method revealed precisely the relationship previously noted. That is, parent-report on each social competence measure positively related to parent-report Theory of Mind across all models. Thus, what appeared to be an informant discrepancy effects using raw scores was actually the result of how closely parent-reported Theory of Mind measures were linked with parent-reported social skills. Specifically, parents who viewed their children as having more skilled Theory of Mind also viewed them as more socially competent. While perhaps intuitive, this is still an important effect to identify, and which has been rarely studied directly in the field [31]. It is also notable that clinician-reported autism severity related to behavioral Theory of Mind, but *not* parent-report of Theory of Mind in all models, suggesting that task performance may be tapping an element of autism presentation that is evident under close scrutiny, but does *not* translate to real-world perspective-taking behavior. Social skill performance in autism mediates some measures of social outcomes such as friendship, inclusion, and belongingness; however, there are subtle aspects of social communication that current task-based performance tests may not adequately pick up on. For example, several studies describe social communication differences and thin slice biases that are present with autistic people [36, 37]. Autistic individuals’ social behaviors frequently produce similar and equally valuable social outcomes when interaction is between autistic people [38, 39]. This is despite these behaviors and ways of interacting being different from those of nonautistic individuals and as such task-performance-based metrics (generally speaking, designed by nonautistic individuals and reflecting nonautistic norms) may not be accurately capturing outcomes that they are intended to capture or elucidate. This highlights the limits of the utility of Theory of Mind to explain the social presentation of autism, and vice versa, particularly in the presence of behaviors such as camouflage and compensation [40] as it is possible—but by no means certain—that camouflaging behaviors are affecting task-based performance. These behaviors—consciously or unconsciously altering one’s presentation, speech, and behavior in order to appear more like nonautistic peers or to compensate for difficulties in communication related to autism—frequently confound less subtle assessments of autism-related communication differences. Autistic individuals frequently engage in deliberate study and practice of social behaviors, norms, rules, and ways of interaction; this deliberate practice does not erase the social differences that autistic individuals often experience, but allows many autistic individuals to fulfill normative roles and expectations, though often not without stress and difficulty. Polynomial regression also revealed a quadratic relationship between parent-report social skill as measured by SSiS-RS and parent-reported Theory of Mind. When parent-reported social skill measured by SSiS-RS was low, an increase in parent-reported social skill had a greater positive relationship with parent-report Theory of Mind (i.e., the slope was steeper) than when parent-reported social skill was high. In other words, at low levels of parent-reported social skills, there was a stronger relationship with parent-reported Theory of Mind. This may be because, when parents observe their autistic children to more overtly struggle socially, they are either more attuned to or concerned about the other challenges (e.g., social cognitive difficulties) they may face. Previous studies have reported that autistic children with poorer parent-reported Theory of Mind also have poorer performance on parent-reported measures of adaptive and social skills [31, 41]. As parents observe their children to be more socially skilled, they may, in turn, become less attuned to (or worried about) the other social cognitive challenges they may face.

Lastly, there was also a quadratic effect of participant-rated social skill as measured by the SSiS-RS on task-based Theory of Mind. At low *and* high participant-rated social skill (as measured by the SSiS-RS), task-based Theory of Mind performance was poor. This may be due to the positive illusory bias truly being evident for *some* autistic youth—specifically, those who report themselves to have excellent social skills. That is, for some autistic youth, their self-report was consistent with social skills challenges supported by their task-based performance. However, other autistic youth viewed themselves as highly skilled while exhibiting similar task-based theory of mind challenges. It is possible that these very difficulties with perspective-taking may lead these youth to miss social cues that would allow them to more accurately rate their social skills. Thus, in this case, behavioral data can help illuminate potential reasons for the large discrepancy between self-report and a task-based approach. Indeed, such youth did exhibit poorer social perspective-taking (Theory of Mind), as hypothesized here and elsewhere in the literature. However, these results add important nuance to this effect, as they appear only evident for a *subset* of the population. On the other end of the self-report social skills scale, autistic youth who reported themselves to have very *poor* Theory of Mind *also* had poor Theory of Mind. This could be attributable to a negative bias extending from negative self-perception [42, 43], it is also possible that, for some, this reflects an accurate self-perception. Conversely, at the middle of the scale, self-perceived social skills were *unrelated* to behavioral Theory of Mind: how youth viewed their social skills was untethered from their ability to understand others’ perspectives. These findings may help to explain the heterogeneity of Theory of Mind in autism, and point towards subgroups wherein social self-perception is (or is not) an effective proxy for perspective-taking. It is also possible that, for some, this reflects an accurate self-perception. Conversely, at the middle of the scale, self-perceived social skills were *unrelated* to behavioral Theory of Mind. That is, how youth viewed their social skills was untethered from their ability to understand others’ perspectives. These findings may help to explain the heterogeneity of Theory of Mind in autism and point towards subgroups in which social self-perception may not be an effective proxy for perspective-taking. Thus, these subgroups may also have distinct clinical needs and be best served by different types of interventions.

## Future Directions

Social informant discrepancies are a fairly well-replicated finding in autistic youth [44–46]. However, our results suggest that these social informant discrepancies may not directly relate to Theory of Mind. Future studies should seek to better understand when social informant discrepancy in autistic youth *does* exist, and how it arises. In addition, future studies should continue to explore the complex relationship between social functioning and Theory of Mind, leveraging both contemporary analytic methods and more naturalistic task-based or clinician-administered Theory of Mind measures. Further, it is notable that there was low correspondence between task-based and questionnaire measures of Theory of Mind. It is possible that either atypical cognitive processes or conscious compensation allow some autistic adolescents to perform well on these Theory of Mind measures, yet are not sufficient for the demands of interaction with non-autistic peers and other individuals in their environment [47, 48]. In many cases, autistic adolescents are capable of performing on par with non-autistic adolescents on explicit, task-based measures of Theory of Mind [49, 50]. However, autistic adolescents perform more poorly on implicit, more naturalistic measures of theory of mind [50–52] and score more poorly on parent-reported measures of Theory of Mind [17, 41]. This may account for the relatively larger effects of parent-reported measures of social skill, social competence, and close friendship on parent-reported Theory of Mind compared to the effects of child-report measures of social skill, social competence, and close friendship on parent-reported Theory of Mind.

## Limitations

This study was subject to several limitations that constrain its interpretation. First, this sample primarily consisted of primarily white, English-speaking middle- and high-income families. This may limit the generalizability of these findings to a less wealthy and more diverse population. Second, this study sample also excluded youth with IQ < 70, excluding youth with a co-occurring intellectual disability. This limits the generalizability of these findings, as autistic youth with co-occurring intellectual disability account for a non-negligible portion of the population of autistic individuals [53]. Third: Our sample was mostly male; autism may manifest itself differently in women and girls and further research is needed in order to see if our observations generalize to a more diverse population [54]. Fourth, this study did not directly address the presence of other co-occurring conditions in the sample; given that conditions such as ADHD are both disproportionately prevalent in autistic youth and can affect social informant discrepancies [55], future work should be sure to consider this and other conditions as potential moderators of the informant discrepancy-Theory of Mind relationship. Lastly, Theory of Mind itself may only be moderately related to other key lived social outcomes in autism, such as peer relations and prosocial behavior, autistic traits, social skills, and empathy and emotional understanding [3], which, in turn may limit the utility of Theory of Mind as a comparator social cognitive construct.

## Summary

This study was the first to utilize polynomial regression analysis as a means of comparing social informant discrepancies and Theory of Mind in a sample of autistic adolescents. This study replicated the presence of positive illusory bias in autistic youth, which was larger than the discrepancy seen in a comparison group. This was driven by autistic participants rating themselves comparably to non-autistic participants, while parents of autistic participants rated their children’s skills approximately one standard deviation lower than parents of non-autistic participants. However, contemporary analytic approaches revealed that this discrepancy was not in fact related to Theory of Mind. In addition, parent-report Theory of Mind and parent-report social functioning measures were linked, while effects of task-based Theory of Mind and social functioning measures were small and related only to a participant-report measure. This indicates that any informant discrepancy that raw differences show may be illusory and must be examined using more sophisticated, modern methods. This study also demonstrates the clinical value of using multiple informants and multiple approaches to measuring Theory of Mind, as it can help to rule out (or rule in) specific hypotheses about factors influencing the social challenges and strengths autistic and other individuals may experience.
